# Accelerated Wound Closure of Deep Partial Thickness Burns with Acellular Fish Skin Graft

**DOI:** 10.3390/ijms22041590

**Published:** 2021-02-04

**Authors:** Randolph Stone, Emily C. Saathoff, David A. Larson, John T. Wall, Nathan A. Wienandt, Skuli Magnusson, Hilmar Kjartansson, Shanmugasundaram Natesan, Robert J. Christy

**Affiliations:** 1 Burn and Soft Tissue Injury Research Department, US Army Institute of Surgical Research, JBSA Fort Sam Houston, Houston, TX 78234, USA; emilycsaathoff@gmail.com (E.C.S.); david.a.larson107.ctr@mail.mil (D.A.L.); johntwall4@gmail.com (J.T.W.); shanmugasundaram.natesan.ctr@mail.mil (S.N.); robert.j.christy12.civ@mail.mil (R.J.C.); 2 Comparative Pathology Department, US Army Institute of Surgical Research, JBSA Fort Sam Houston, Houston, TX 78234, USA; nathan.a.wienandt.mil@mail.mil; 3 Kerecis^®^, 101 Reykjavik, Iceland; sm@kerecis.com (S.M.); hkj@kerecis.com (H.K.)

**Keywords:** swine, burn, epithelialization, contraction, fish skin graft, fetal bovine dermis, cellular and tissue-based product

## Abstract

Thermal injuries are caused by exposure to a variety of sources, and split thickness skin grafts are the gold standard treatment for severe burns; however, they may be impossible when there is no donor skin available. Large total body surface area burns leave patients with limited donor site availability and create a need for treatments capable of achieving early and complete coverage that can also retain normal skin function. In this preclinical trial, two cellular and tissue based products (CTPs) are evaluated on twenty-four 5 × 5 deep partial thickness (DPT) burn wounds. Using appropriate pain control methods, DPT burn wounds were created on six anesthetized Yorkshire pigs. Wounds were excised one day post-burn and the bleeding wound beds were subsequently treated with omega-3-rich acellular fish skin graft (FSG) or fetal bovine dermis (FBD). FSG was reapplied after 7 days and wounds healed via secondary intentions. Digital images, non-invasive measurements, and punch biopsies were acquired during rechecks performed on days 7, 14, 21, 28, 45, and 60. Multiple qualitative measurements were also employed, including re-epithelialization, contraction rates, hydration, laser speckle, and trans-epidermal water loss (TEWL). Each treatment produced granulated tissue (GT) that would be receptive to skin grafts, if desired; however, the FSG induced GT 7 days earlier. FSG treatment resulted in faster re-epithelialization and reduced wound size at day 14 compared to FBD (50.2% vs. 23.5% and 93.1% vs. 106.7%, *p* < 0.005, respectively). No differences in TEWL measurements were observed. The FSG integrated into the wound bed quicker as evidenced by lower hydration values at day 21 (309.7 vs. 2500.4 µS, *p* < 0.05) and higher blood flow at day 14 (4.9 vs. 3.1 fold change increase over normal skin, *p* < 0.005). Here we show that FSG integrated faster without increased contraction, resulting in quicker wound closure without skin graft application which suggests FSG improved burn wound healing over FBD.

## 1. Introduction

According to the American Burn Association’s National Burn Repository, the annual burn incidence rate is approximately half a million people in the United States (US). This includes approximately 40,000 hospitalizations as a result of burn related injuries and 3400 deaths in the US each year [1]. Currently, the standard of care for the treatment of deep partial thickness (DPT) and full thickness (FT) burns involves wound excision followed by early coverage with autologous skin grafts or flaps [2]. In many cases [i.e., those where there is a large total body surface area (TBSA) burn], there is limited donor site availability to obtain autografts for reconstruction [3]. Even if available, autologous skin grafts or soft tissue flaps often achieve a less than ideal outcome, which includes significant donor site morbidity [4]. Many burn patients endure multiple surgical interventions for years after their initial injury, which significantly delays their recovery and return to normal function [5]. The gap in achieving satisfactory results lies in the inability of current technologies to assist the patient in producing and maintaining durable skin which resists the forces of contraction [6,7]. In short, there is a great need for off-the shelf products such as cellular and tissue-based products (CTPs) that could be used instead of grafts, which incorporate into healing tissue, improve cosmetic outcome, and circumvent donor site morbidities [8].

A wide variety of CTPs are currently available to treat wounds of a myriad of etiologies in the US via the 510 k Food and Drug’s Administration (FDA) designation [9]. CTPs are sourced from a number of animals but are not necessarily skin derived [9]. Fetal bovine dermis (FBD), for example, is a dermal repair scaffold that has been indicated in the treatment of complex wounds, including full thickness wounds and chronic diabetic ulcers [10]. FBD is uniquely rich in type III collagen, a fibrillar collagen that is active in wound healing and in developing tissues [11]. Following rehydration, FBD exhibits both strength and pliability, making it an ideal extracellular structure for tissue regeneration and revascularization [12]. Acellular fish skin grafts (FSG) are a CTP that are created by minimally processing fish skin from the Atlantic cod (*Gadus morhua*). Interestingly, there are no known prion, bacterial, or viral diseases that can be transmitted from North-Atlantic cod to humans; hence, the minimal processing requirements [13]. FSG is more similar in structure to human skin than anti-viral-processed skin substitutes such as amniotic membrane [14] and other CTPs [15]. The FSG is processed using a proprietary method that preserves the structure and lipid composition of the skin [14]. FSG is also rich in Omega3 fatty acids, which possess multiple health benefits [16]. Applications include: burn skin reconstruction; chronic and oral wound; hernia repair; breast reconstruction; and dura mater reconstruction. A double-blind, comparative, randomized control trial (*n* = 81) indicated FSG favorably compared with mammalian CTPs in wound closure [17]. When grafted onto damaged human tissue such as a diabetic wound, the host cell response to the applied material facilitates regeneration of new living tissue [16,18]. Both CTPs evaluated in this study are FDA approved, available in multiple sizes and meshed ratios, and according to each company’s website are both in the high cost group for Medicare National Payment rates for application of skin substitutes.

This study was conducted with the objective of comparing FSG to a mammalian CTP (fetal bovine dermis, FBD) and evaluating FSG treatment of DPT burn wounds through the utilization of a preclinical porcine burn wound model. We are testing the FSG product to determine how well it works as a CTP on DPT burn wounds, how well it will integrate into the wound bed, and if it will improve long term healing.

## 2. Results

### 2.1. Fish Skin Graft Integrated into the Wound Bed Faster than Fetal Bovine Dermis

**Figure 1:**
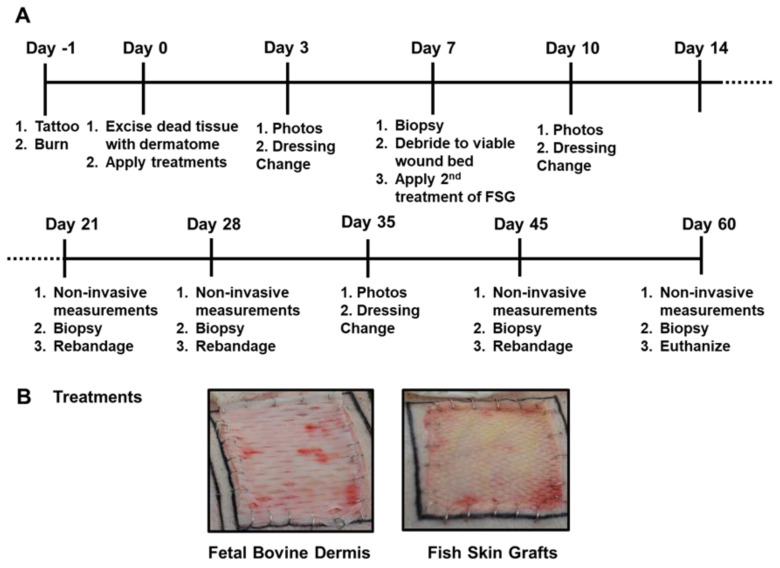
Porcine Burn Wound Experimental Timeline and Treatments. (**A**) The timeline is depicted for the 62 day study with the associated procedures performed throughout the study. Non-invasive measurements included digital and laser speckle imaging, TransEpidermal Water Loss (TEWL), and hydration readings. (**B**) Representative digital images of both fetal bovine dermis (FBD) and fish skin graft (FSG) after application on Day 0.

**Figure 2:**
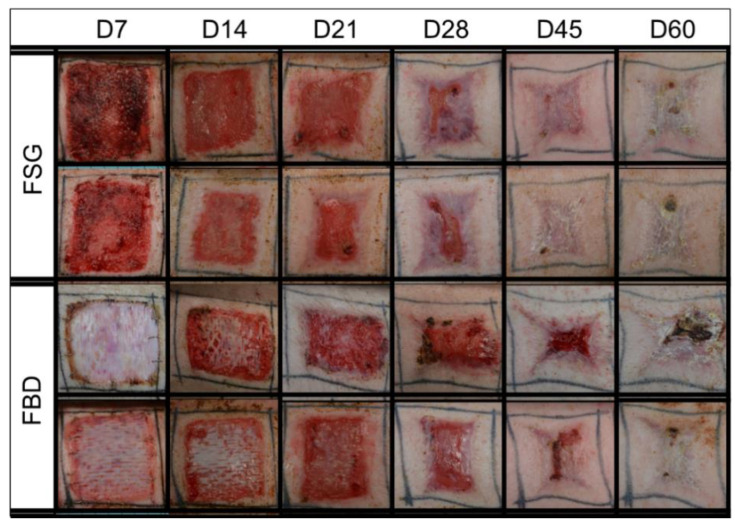
Representative Digital Images. Digital images shown are examples from 2 of the animals in the study. These were captured of all wounds during the 62 day study and were utilized to calculate the re-epithelialization and contraction rates. Biopsy punches harvested on previous time points are visible as small scabs in some wounds. Fetal bovine dermis (FBD); Fish skin graft (FSG).

The timeline followed over the course of the 62 day experiment is illustrated in Figure 1A,B shows representative day 0 images with wounds treated with meshed CTPs. Both CTPs were easy to apply to the excised burns and no infection was observed in any wound. As shown in Figure 2, the 1st application of FSG resulted in a highly granulated wound bed by day 7 in which observable seroma formed under the FSG in both of the wounds on the 1st animal. The FSG is meshed at a 1:1.5 ratio and for all subsequent treatments, extra precaution was made to ensure the product was fully expanded during application by additional staples at the wound edge. For the remaining 10 wounds treated with FSG, only 1 additional seroma was observed by the day 7 time point. The 2nd application of FSG quickly integrated and turned translucent on the wound. The FBD was easy to identify in the wound bed due to its opaque appearance and was seen as late as day 21 in some wounds. Supplemental Figure S1 shows the complete blood count (CBC) results and indicates all values were in the normal range for pigs.

### 2.2. Wound Closure Rate Was Improved by Application of Fish Skin Graft

**Figure 3:**
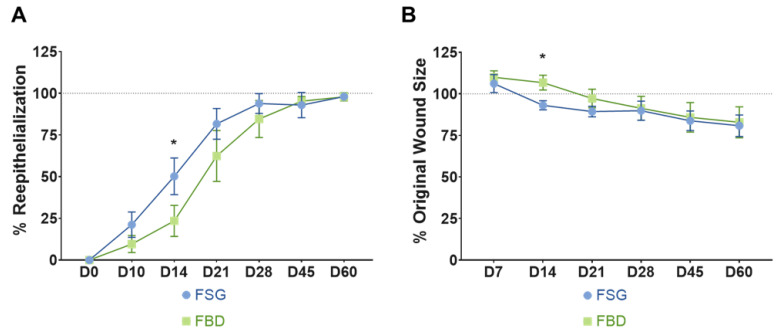
Wound Closure rates. (**A**) Re-epithelialization was calculated by tracing the leading edge of the epidermis and comparing to total wound size. The dotted line represents 100% wound re-epithelialization. No day 7 results could be determined for re-ep due to the reapplication of FSG to the wounds which made observing the wound edge not possible. (**B**) Wound contraction was calculated by tracing the tattoos, normalizing to the growth of each animal, and comparing to the initial wound size. The dotted line represents the original wound size. Any reduction below that line is the result of the wounds contracting as they heal. For both A and B) * = *p* < 0.05 comparing treatments via a 2-way repeated measures ANOVA with Sidak’s post-hoc test (*n* = 12). Fetal bovine dermis (FBD); Fish skin graft (FSG).

Wound closure was assessed by two methods: determining the 1) re-epithelialization and 2) contraction rates. As quantified in Figure 3A, the wounds treated with FSG resulted in faster re-epithelialization beginning at day 10 until day 28; however, this was only significant at day 14 when compared to FBD (50.2% vs. 23.5%, *p* < 0.005). The contraction rates are reported as % original wound size (Figure 3B) and a significant reduction in original wound size at day 14 was observed for the FSG when compared to FBD (93.1% vs. 106.7%, *p* < 0.005, respectively). This difference did not continue and beginning at day 28 the wounds contracted at similar rates regardless of treatment.

### 2.3. Functional Skin Measurements Nearly Returned to Normal Levels by Day 60

**Figure 4:**
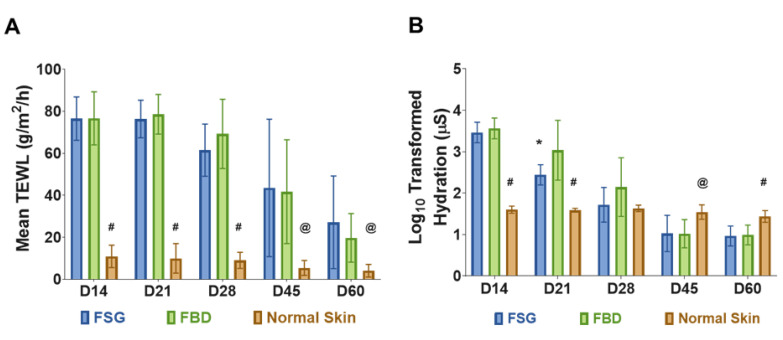
Skin Barrier Function Measurements. (**A**) TransEpidermal Water Loss (TEWL) measures the barrier properties of the epidermis. At each time point, three TEWL measurements were taken for each of the twenty four wounds and then averaged. (# = *p* < 0.0001; @ = *p* < 0.05 for normal skin vs. treatment groups). (**B**) Hydration levels of wounds on follow-up days represented in Log scale (a log transformation was performed on hydration data because measurements ranged from 10 to 10,000). The hydration measures the water content of the wounds. Five measurements were obtained for each wound at each time point and averaged. (# = *p* < 0.001 and @ = *p* < 0.05 for normal skin vs. CTPs; * = *p* < 0.05 for FBD vs. FSG). For A and B, significance was detected using a 2-way ANOVA mixed effects model and a Tukey post-hoc test. Fetal bovine dermis (FBD); Fish skin graft (FSG).

Restoration of normal skin function was assessed by measuring TEWL and hydration levels of the wounds. No differences in TEWL measurements were observed between the two treatment groups. Since the wounds were allowed to heal by secondary intentions, significantly higher water loss values were observed throughout the experiment comparing the CTPs to the normal skin (Figure 4A). Similarly, the hydration values were significantly higher in the CTPs compared to normal skin but only for the day 14 and 21 time points (Figure 4B). However, by day 28 wounds treated with CTP remained hydrated close to the levels of normal skin without any significant difference. As the wounds re-epithelialized, the hydration values decreased and were significantly lower at the day 45 and 60 time points. The FSG was also observed to have significantly lower hydration values compared to FBD at day 21 (309.7 vs. 2500.4 µS, *p* < 0.05).

### 2.4. Fish Skin Graft Treatment Resulted in an Early Increase in Blood Flow

**Figure 5:**
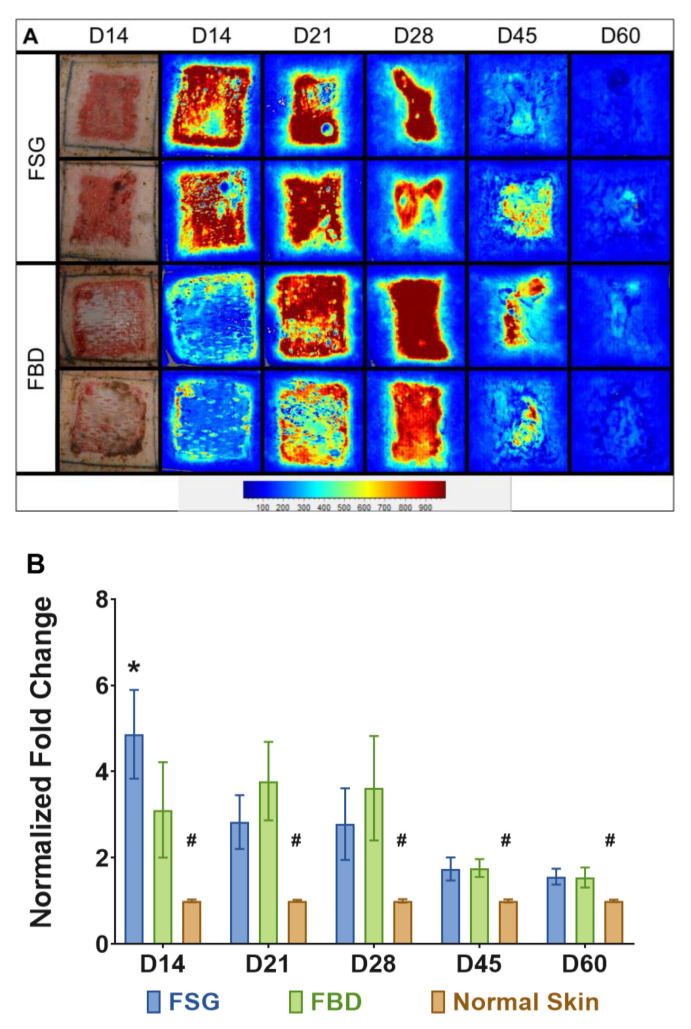
Laser Speckle Imaging. (**A**) Two representative wounds are shown for both treatment groups. Digital images at day 14 and the corresponding laser speckle images (LSI) throughout the experiment are shown. The heatmap scale is shown below in which blue represents low perfusion and red if highly perfused. Note that the perfusion in uninjured skin is a sky blue and as the wounds heal by day 60, the borders of the wound are no longer obvious. (**B**) Quantitation of LSI measurements represented as a fold change above the normal perfusion around each wound and normalized to the growth controls on each animal at the designated time point (# = *p* < 0.005 for normal skin vs. treatment groups; * = *p* < 0.05 for FSG vs. FBD as determined by 2-way repeated measures ANOVA with Tukey post-hoc test, *n* = 12). Fetal bovine dermis (FBD); Fish skin graft (FSG).

Figure 5A are the illustrative images representing blood flow on the wound bed treated with CTPs, observed throughout the experimental time-line. The light blue regions represents the basal amount of blood flow in normal skin surrounding the wound bed, whereas the wound bed appears dark red as the perfusion reaches a maximum. At day 14, the FSG images are mostly red within the wound while the FBD are still mostly blue as the LSI is not able to detect blood flow if the product has not integrated well (Figure 5A). Similar intensities in the images are observed at the other time points. Quantitative analysis confirms this trend and significantly higher blood flow was measured for FSG at day 14 via laser speckle (4.9 vs. 3.1 fold change increase over normal skin, *p* < 0.005) (Figure 5B). It should be noted that significantly higher values were observed throughout the experiment when comparing the CTPs to the normal skin.

### 2.5. Histological Analysis

**Figure 6:**
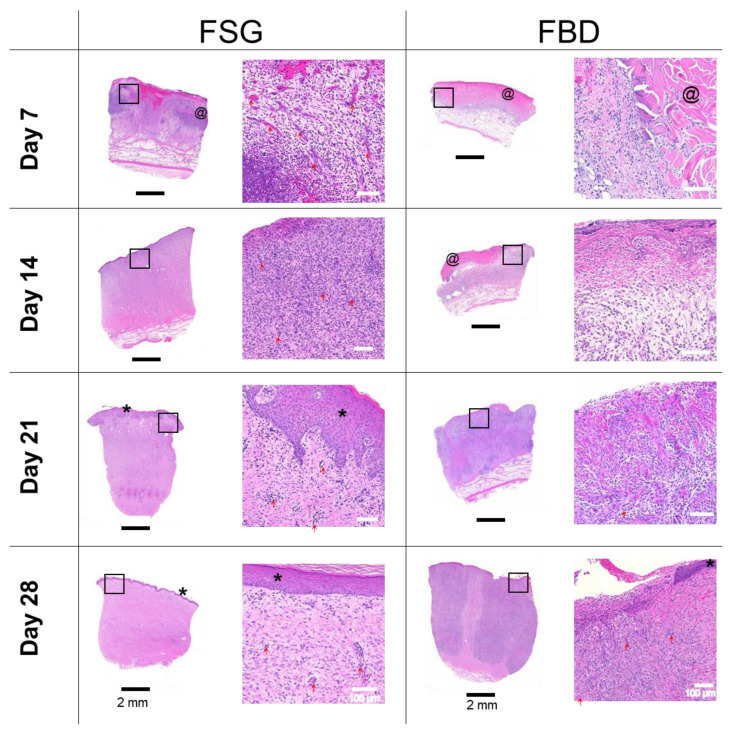
H&E Histology. Representative H&E images of biopsy punches harvested at the indicated time points. Images on left are the entire biopsy punch with black scale bars = 2 mm while the area indicated by the black box is the enlarged image on the right with white scale bars = 100 um. * = newly formed epidermis; @ = residual pieces of treatment in wound bed; red arrows are pointing out some of the newly formed blood vessels. Fetal bovine dermis (FBD); Fish skin graft (FSG).

**Figure 7:**
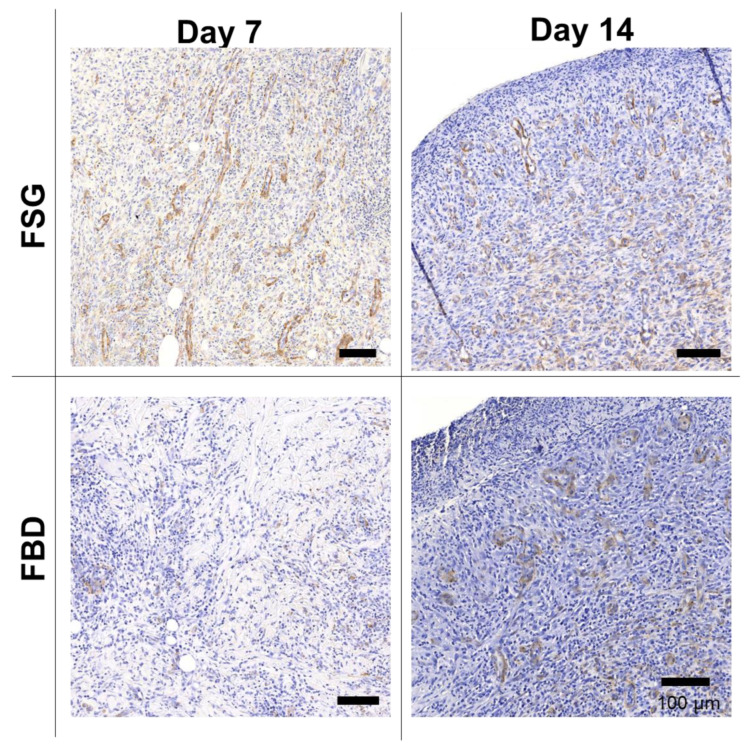
Immunohistochemistry with alpha-smooth muscle actin. Representative α-SMA images of biopsy punches harvested at the indicated time points. Positive brown staining is outlining the newly formed blood vessels. Scale bars = 100 um. Fetal bovine dermis (FBD); Fish skin graft (FSG).

Figure 6 shows one representative wound for each CTP treatment group over time stained with H&E. The CTPs are visible in the day 7 images but only FBD is observed at day 14. The immune response is rather prolonged in the FBD treatments as indicated in the images with the darker purple staining areas representing a mixed population of inflammatory cells. The FSG has extensive granulation tissue formation on day 14 and beyond with complete epidermis visible at day 21. A high number of newly formed blood vessels are seen as early as day 7 for the FSG treated wounds while it is hard to discern many in the H&E images. For this reason, sections were stained with α-smooth muscle actin (α-SMA) to help visualize the newly formed vessels. Figure 7 shows a higher number of α-SMA positive stained blood vessels in the FSG treated wounds at day 7 compared to the FBD with both groups showing comparable amounts at day 14.

**Figure 8:**
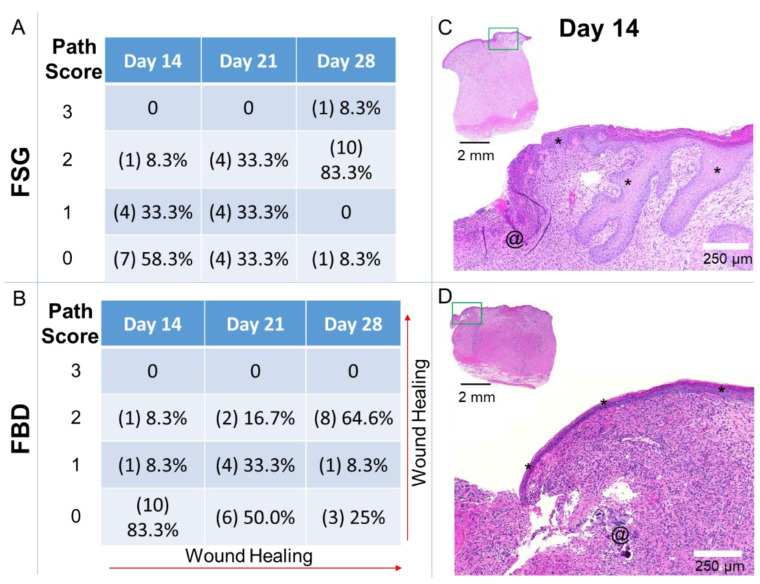
Pathology Scores for Re-epithelialization. Sections were assessed by a Board-Certified Veterinarian Pathologist. (**A**,**B**) The number of slides receiving the designated score are indicated in (#) with the calculated percentages out of the 12 wounds for each treatment at each time point. Path Scores: 3 = Normal epidermis across entire wound bed; 2 = regenerating or hyperplastic with 100% coverage; 1 = partial epidermis; 0 = no epidermis. (**C**,**D**) Day 14 example of hematoxylin and eosin stained sections with a path score of “1” with the upper left image showing the entire biopsy punch. The area outlined with a green box is magnified and shown in the image to the lower right with scale bar = 250 µm. * = newly formed epidermis; @ = residual pieces of treatment in wound bed. Fetal bovine dermis (FBD); Fish skin graft (FSG).

Histological sections were scored by a Board-Certified Veterinary Pathologist, blinded to the study treatment groups. The pathological results are represented in Figure 8 and Supplemental Table S1. The histological increase in the re-epithelialization rates following FSG treatment corroborated to our quantitative epithelialization measurements reported in Figure 3A. The pathology analysis (Figure 8) further showed that the wounds treated with FSG had 5/12 and 8/12 with partial to 100% epidermis compared to only 2/12 and 6/12 for FBD at day 14 and 21, respectively. The most notable difference was observed in the day 28 sections with 11/12 FSG and only 8/12 FBD treated wounds resulting in complete epidermal formation.

Supplemental Table S1 indicates little (path score 1) to no (path score 0) granulation tissue formation in 8/12 FBD treated wounds at Day 7 compared to 0/12 FSG. The presence of foreign material was observed to persist at days 14, 21, and 28 in 10/12, 5/12, 2/12 FBD treated wounds (path score 1) compared to 5/12, 0/12, 0/12 FSG treated wounds, respectively. The abundance of newly formed vessels (path score 2 or 3) was assessed with 8/12 and 8/12 FSG compared to 0/12 and 3/12 FBD observed at day 7 and 14, respectively.

## 3. Discussion

Current strategies to improve burn wound healing outcomes and reduce the need for donor sites mainly include synthetic and biological skin replacements products [19,20]. In particular, the Army’s future multi-domain operations recognizes the need of materiel products to treat severe burns that are compatible with existing pre-hospital care being used by the military in austere, combat environments. These wound dressings are expected at the point of injury to provide antimicrobial protection and temporary coverage for a prolonged period of time. In this study, two types of acellular skin grafts derived from a fish skin rich in omega-3 fatty acid and a fetal bovine dermis were evaluated in surgically debrided DPT burn wounds, randomized over six anesthetized Yorkshire pigs. Both CTP treatments successfully provided temporary wound coverage and led to the formation of granulated wound beds suitable for the application of subsequent skin grafts, if that definitive therapy would have been desired. With both treatments indicated for the healing of deep partial thickness burns, overall wound healing measures with FSG was compared with FBD in a preclinical porcine burn wound model. In accordance to the Wound-care Experts/FDA-Clinical Endpoints Project and within the context of recommended endpoints listed as the highest priority for exploration in the research phase of the project for evidence supporting FDA acceptability for use as clinical outcomes assessments, we chose wound closure, which is a combination of epithelialization and contraction, as the primary end endpoint [21]. In addition, we assessed wound vascularization, hydration, and restoration of barrier functions as secondary measures to establish functional wound healing outcomes following the treatment with FSG and FBD. FDA recognizes success of an off-the shelf treatment as complete wound closure, defined as skin re-epithelialization without drainage or dressing requirements confirmed at two consecutive study visits 2 weeks apart [22]. It is important to note that human wounds don’t heal with the same timeline as animals and our results may not be generalizable to the clinical situation. In our study, a near-complete (>90%; Figure 3A) reepithelization of DPT burn wounds occurred with two FSG applications, and within 4 weeks of injury and initial treatment. FSG exhibited faster rate of re-epithelialization without an increase in contraction compared to the other commercial comparator (FBD) used in this study. Quicker reepithelization with FSG became statistically significant at day 14 and indicates FSG’s potential as a treatment well-suited for large wound closure. Although both FSG and FBD resulted in full reepithelization by day 60 post burn, the findings of this study are clinically significant in that accelerated wound closure allows for less time for potential infection to occur. These findings suggest that the application of FSG on large DPT burn wounds could potentially be successful in enhancing wound closure while also eliminating future infection scenarios and any associated morbidities.

Minimal processing may have contributed to the accelerated wound closure by FSG due to the preservation of bioactive compounds that support cell in-growth, particularly omega-3 polyunsaturated fatty acids. Previous studies have shown that omega-3 polyunsaturated fatty acids are characteristic of FSGs and possess anti-inflammatory effects that have proven beneficial for faster wound healing, though further studies are required to fully understand the mechanism through which this is accomplished [13,14,17,23]. In a non-inferiority study, 81 people volunteered to undergo 2 full thickness 4-mm biopsy punches on their arm approximately 2 cm apart and the wounds were treated with FSG or porcine small-intestine submucosa (a CTP). The participants had weekly follow-up visits for 28 days resulting in no difference for the primary end point (95% vs. 96% of wounds healed) [17]. Nevertheless, we observed subsidence of neutrophils after 3 weeks in FSG treated wounds, paralleling formation of near-complete epithelia. Whereas, FBD treated wounds were still infiltrated with neutrophils even at 4 weeks. Further, there was an ominous presence of macrophages throughout the course of observation in FBD treated wounds, indicating wounds are still in a state of active inflammation for a prolonged period of time. Meanwhile, the FSG treatment caused subsidence of macrophages after 3 weeks. In agreement with the wound healing phase, FSG treatment instigated macrophages early recruitment sustaining till day 14, and decreased post day 21, indicating wound perhaps switched to progressive remodeling phase, and aid in collagen reorganization and maturation [24,25].

Other laboratories have reported similar time frames (20–28 days) for DPT burn wounds to heal by secondary intentions (without a skin graft). Wang et al. used larger burns than ours (~50 cm^2^ vs. 25 cm^2^) and reported 80% re-epithelialization occurring after 20 days for caudal burns and 25 days for cranial sided burns [26]. Burmeister et al. used smaller burns than ours (~7 cm^2^ vs. 25 cm^2^) and reported 94% re-epithelialization on day 28 assessed histologically via biopsy punches [27]. Faster reepithelization has been associated with significantly greater early time-point contraction in previous experiments, but the quicker reepithelization rates observed using FSG did not result in higher contraction rates in this study. Specifically, in those previous studies, re-epithelialization occurred faster with higher contraction as shown in Supplemental Figure S2. Faster re-epithelialization was observed in the control group with slightly higher contraction rates when comparing FSG and FBD to a historical control group that received no CTP as a treatment but standard dressings only (similar bolster using antimicrobial Telfa and gauze) on the same size burns (5 × 5 cm), dermatome surgical debridement, and dressings changes from 5 previous animals (Supplemental Figure S2A,B). This suggests applying a CTP to the wound bed may reduce the mechanical forces and therefore decrease contraction [28,29]. These historical controls have illustrated the importance of measuring multiple early time-point parameters (day 14), including contraction and re-epithelialization. By 28 days, all wounds had contracted significantly, and no statistically significant differences remained. Thus this study aimed its focus at the effects of both contraction and reepithelization on overall wound closure. Wound closure that takes place at a rate faster than that of dermal matrix remodeling can lead to extensive scarring for patients with severe burn wounds. However, the early less wound contraction observed on day 14 in this experiment for wounds treated with FBD is likely a result of FBD taking longer to fully integrate into the wound bed and possibly providing a mechanical barrier resistant to contractile forces. Faster integration time was observed for FSG treated DPT burn wounds compared to FBD. The FSG product was easy to apply but there were difficulties associated with primary and secondary dressings, indicating that further optimization is needed to prevent product removal at dressing changes. Both CTP treatments led to the formation of mechanical barriers capable of minimizing contractile forces within the wounds. The combination of accelerated wound closure and lessened contraction for wounds treated with FSG is indicative of FSG’s potential for decreasing likelihood of both infection and scarring. A limitation of this study was that scarring was not directly assessed and we can only infer the CTPs impact from the contraction results. An appropriate model for hypertrophic scarring would be to utilize Red Duroc swine that is well characterized and accepted in the burn community [30,31,32,33]. In addition, extending the length of the study to 90 or 120 days and assessing the wounds specifically for scarring by a modified patient and observer scar assessment scale (POSAS) or Vancouver scar scale, erythema, melanin, picrosirius red, contraction, and viscoelastic properties (elasticity, firmness, tonicity, and suppleness) would be recommended for any future experiments.

An unexpected finding was the formation of seromas on two animals with DPT burn wounds that were treated with FSG, though this is possibly related to FSG being meshed 1:1.5 whereas FBD was meshed 3:1. A wider meshed graft would allow for greater expansion of FSG over the wound bed which would perhaps prevent seroma formation from occurring. It is unclear why the second treatment of FSG did not exacerbate this issue. An alternative explanation is that seroma formation is the result of leakage from hypergranulating tissue present between the wound bed and graft. This is supported by the observation of an early immune response for wounds treated with FSG in contrast to a delayed immune response until day 21 for those treated with FBD. Corroborating to this finding, hemorrhage severity scores were higher in later time points in wounds treated with FBD.

To this end, an effective CTP is one that is able to successfully induce wound closure and restore barrier function of injured tissue as quickly as possible while minimizing infection, scarring, and contracture. In this study, the long term healing of DPT burn wounds was similar for both FSG and FBD when allowed to heal via secondary intentions. No differences were observed in TEWL measurements between FSG and FBD, but this was unsurprising given that the length of the experiment allowed for epidermis reformation and subsequently barrier function restoration as well. The lack of any statistically significant differences in TEWL measurements for the two treatments indicates normal reformation of the epidermis. On the contrary, notable differences in the hydration levels of FSG and FBD treated DPT burn wounds compared to normal skin levels were observed. A possible explanation for the large range in data is that no moisturizers were applied to the wounds at any point throughout the experiment, which might account for the wounds being particularly dry (below normal) by day 60. It is a common practice in continuing care of patient with a burn injury to soothe the burn area with an emollient to maintain moisture and hydration [34]. Small sample sizes (6 animals with 12 total wounds for each treatment) may also contribute to any data disparities. Hydration levels of FSG treated DPT burn wounds correlated closely with the reepithelization rates for FSG, with levels returning to that of normal skin function faster with FSG treatment (day 21). Similarly, a pronounced perfused wound bed was observed via laser speckle on day 14 whereas results indicate it took FBD treated wounds approximately 21 days to fully integrate. Overall, the long term results for FSG and FBD treated DPT burn wounds was similar, but the faster integration of FSG into the wound bed could prove clinically advantageous in scenarios where autografting is not a possibility.

In anticipation of conflict with near-peer adversaries, military focus has shifted to the emerging challenge of providing prolonged field care (PFC) in the operational environment. PFC aims to provide advanced treatment capabilities farther forward in the continuum of care in the event that the golden hour for evacuation is no longer feasible [35]. For PFC, surgical debridement is not currently possible, but non-surgical debridement (NSD) is being explored to determine the optimal method for removing the necrotic tissue [36]. If this is achieved, a need exists to identify the best off-the shelf product that will work in conjunction with NSD to temporize the burn wounds to allow spontaneous healing and eliminate the need for a skin graft or act as a bridge until definitive care can be provided. The future multi-domain battlefield will require prolonged and en-route care within and from theater with delays of days up to a week for severely burned patients being expected [37]. Should battlefield conditions not allow for the removal of the necrotic tissue in a timely manner, the exposed wound will remain unprotected and a greater risk of infection and delayed healing will occur. Rapid fielding of products closer to the point of injury is essential and can provide temporary burn wound coverage for longer time periods when delayed evacuation exceeds available capability prior to definitive care [38]. The ultimate goal is to identify the best solution to heal wounds so that injured service members may not need evacuation to the burn clinic and combat power can be preserved close to the area of operation.

In this study, FSG demonstrated properties characteristic with those of normal skin functioning and promoted accelerated granulation tissue formation for DPT burn wounds. The FSG product undergoes minimal processing, is easy to apply, and accepted by more cultures than other CTP products on the market, making it well-suited for use in clinical settings. In particular, FSG fits well within the scope of Army’s mission to fill the capability gap in Service Members burn treatment and identify products that may reduce or replace the use of autologous and allogeneic skin grafts for the management of burn wounds requiring surgical excision. However, the use of a preclinical porcine model makes it difficult to predict how these findings will translate to wounds on human skin. In the future, studies should aim to employ appropriate standard dressings control groups and final wound assessments for scarring on the appropriate strain of animals to assess the differences between CTP treatments. Additionally, FSG’s enhanced healing properties also make it an ideal candidate for large TBSA burn wound treatment. Studies exploring this concept and comparing the inflammatory mechanisms of FSG and FBD will allow for a better understanding of their effectiveness as respective CTPs in place of skin grafts.

## 4. Materials and Methods

### 4.1. Animals

Research was conducted in compliance with the Animal Welfare Act, the implementing Animal Welfare Regulations, and the principles of the Guide for the Care and Use of Laboratory Animals, National Research Council. The U.S. Army Institute of Surgical Research (USAISR) facility’s Institutional Animal Care and Use Committee (IACUC) approved all research conducted in this study (IACUC animal protocol A-16-021-TS5 approved 5 May 2017). The USAISR, where this research was conducted, is fully accredited by AAALAC. This study utilized six female Yorkshire pigs (Midwest Research Swine, Gibbon, MN, USA) of weights 51.8 ± 3.3 kg at the time of injury. Animals were given a minimum of 7 days to adjust to the facilities prior to the experiment and were able to eat and drink *ad libitum*.

### 4.2. Anesthesia

The animals were anesthetized during each procedure performed throughout the 62 day study, specifically on the 11 days indicated in Figure 1. Each animal was fasted before administration of anesthesia. An intramuscular (IM) injection of glycopyrrolate (0.01 mg/kg) or atropine (0.02 mg/kg) was administered in the neck region of the animals as needed before anesthesia to reduce saliva secretion and prevent the occurrence of vagally mediated bradycardia during surgery. Tiletamine-zolazepam (Telazol, 4–6 mg/kg, IM) or ketamine (10–25 mg/kg, IM) was used to induce the animals and anesthesia was initially administered through 3–5% isoflurane in oxygen via a facemask. The animals were intubated with an endotracheal tube and received automatic ventilation with an initial tidal volume at 8–12 mL/kg, peak pressure at 20 cm H_2_O and respiration rate at 8–20 breaths per minute. Adjustments were made to ventilator settings to help maintain an end tidal PCO_2_ of 40 ± 5 mmHg. Anesthesia was maintained with 1% to 3% isoflurane in oxygen. Analgesia was preemptively applied Day-1 before wounding and again with sustained release buprenorphine (0.1–0.24 mg/kg; Veterinary Technologies/ZooPharm, Windsor, CO, USA) or hydromorphone (0.2–0.6 mg/kg) given subcutaneously (SQ) in either the dorsal lumbar, lateral neck, or caudal hamstring as needed. Venipuncture was routinely used for the duration of the study to harvest blood from the external jugular for complete blood counts (CBC) and biochemistry labs.

### 4.3. Porcine Burn Wounds

Chemical depilation (Nair™, Church & Dwight, Ewing, NJ, USA) was used to remove the hair on the dorsum of the Yorkshire pigs and the site was subsequently rinsed with sterile water. Square tattoos were applied around the perimeter of each wound using an electric tattoo machine. In addition to designating wound number, the permanent markings served as reference points for burn creation, all imaging, and for the calculation of re-epithelialization and contraction rates. On each animal, ten 5 × 5 cm square wounds were created (4 cm from each other and 2–3 cm from spine) on the dorsum evenly distributed in two rows. Four DPT and six FT burns were created on the dorsum of anesthetized animals with a thermocoupled brass burn device heated to 100 °C as described previously [39,40]. Additionally, a force meter was utilized to deliver constant and consistent pressure (~0.4 kg/cm^2^) to all wounds. Each animal was also marked with 2 control regions consisting of unburned normal skin. Across six animals, 24 DPT and 36 FT burns were created. This manuscript is only presenting the results of the DPT wounds treated with CTPs. The results of the FT wounds will be reported in a separate manuscript due to differences in the burn depth and the treatments that were applied to the wounds (i.e., FBD was not applied to FT wounds but FSG was). Images were captured throughout the experiment with a Nikon digital camera and a Silhouette™ (Aranz Medical, Christchurch, New Zealand) hand held 3D laser image capture device. On day of burn, the wounds were covered with sterile nonadherent gauze (Telfa, Kendall, Mansfield, MA, USA) and an antisepitc occlusive dressing (Ioban™, 3M, St. Paul, MN, USA).

### 4.4. Surgical Debridement and Treatment

One day after burning, designated as Day 0, anesthetized animals had dressings removed and their backs sterilized. Surgical debridement was performed by using a pneumatic dermatome (Zimmer, Warsaw, IN, USA) to tangentially remove necrotic burn tissue until a bleeding wound bed was observed. From careful observations made from our other studies (unpublished), two passes of the dermatome set at 0.030” was sufficient to remove most if not all necrotic material for DPT burns on the back of this size Yorkshire pigs. An additional pass set at ~0.010” and/or a curette was used to remove any remaining burned tissue down to the viable wound bed. Two DPT wounds on each animal were treated with FSG (Kerecis^®^ Omega 3 Burn, Kerecis^®^, Reykjavik, Iceland) or FBD (Primatrix™, Integra LifeSciences, Princeton, NJ, USA) for a total of 12 wounds treated with each CTP. These products were rehydrated in sterile saline prior to application to make pliable, cut to fit, and secured to each wound with staples. Wounds were covered with a bolster consisting of antimicrobial Telfa (Kendall, Mansfield, MA, USA) wrapped around sterile gauze. Additional sterile gauze was applied between the bolsters, secured in place with Elasticon (Johnson and Johnson, New Brunswick, NJ, USA), and covered with a fabric vest (DeRoyal, Powell, TN, USA) to protect the treatments from infection and mechanical shearing. This dressing protocol was utilized through the day 14 time point after which the bolsters were no longer necessary. On day 7, a second application of FSG was applied to the previously treated wounds after a gentle wet debridement around the wound periphery.

### 4.5. Wound Care

Once the dressings were removed, the animals’ backs were rinsed with diluted 4% chlorhexidine gluconate (Hibeclens^®^, Molnlycke Health Care, Norcross, GA, USA), sterile water, and patted dry with sterile gauze. In an effort to keep the wounds clean, additional routine wound care was initiated on Day 14. This was accomplished with gentle wet debridement using sterile gauze and water to clean up any wound exudate from the wound periphery.

### 4.6. Re-Epithelialization and Contraction Calculations

SilhouetteConnect™ software (Aranz Medical, Christchurch, New Zealand) was utilized to determine the re-epithelialization and contraction of the wounds over time. The software allows several regions of interest to be generated by tracing and the results are batch exported to Microsoft Excel 2016 (Microsoft, Redmond, WA, USA). The open wound area (*OWA*) of each wound was traced at each time point. To calculate re-ep % at each time point, the following formula was utilized with *D*0 as the initial *OWA* at day 0 and *Dx* representing the *OWA* at the other time points.

(1) $$\text{Re-ep}\%\;=\;\frac{\left[OWA\;\left(D0\right)-OWA\;\left(Dx\right)\right]}{OWA\;\left(D0\right)}*100$$

To determine contraction, the tattoo area was traced around each wound at all-time points. The normalized tattoo (*Dx*) was determined by dividing the area measured at the time point for each wound by the average increase in area measured in the 2 growth control wounds.

(2) $$\%\mathrm{Original}\;\mathrm{Wound}\;\mathrm{Size}\;=\;\left(1-\frac{\left[Tattoo\;\left(D0\right)\;-\;Normalized\;Tattoo\;\left(Dx\right)\right]}{Tattoo\;\left(D0\right)}\right)*100$$

### 4.7. Skin Function Measurements

A DermaLab Combo transepidermal water loss (TEWL) and hydration device (Cortex Technology, Hadsund, Denmark) were utilized to measure skin moisture properties. TEWL measures the density gradient of evaporative water loss from the skin and is expressed in gram per hour per square meter. The hydration probe measures the water binding capacity of the stratum corneum. Three readings were acquired for TEWL (cranial, middle, and caudal) and five for hydration (cranial, middle, caudal, medial, and lateral) and averaged for each wound at each time point. The rationale on only 3 readings for TEWL is that those measurements would take 0.5–2 min each whereas the hydration required less than 10 s per wound.

### 4.8. Laser Speckle

Microcirculation of the injured skin was measured via a high resolution laser speckle imaging device (LSI; Moor FLPI, Moor Instruments Inc., Wilmington, DE, USA), positioned 10–20 cm from each wound. Image acquisition settings were: Gain: 200, Exposure time: 4 ms, Time constant: 1.0 s, Filter: 250 frames, Sample interval: 20 s, Image resolution: 760 × 568. To achieve image analysis, all frames obtained were merged together into one image. Three regions of interest were drawn including one area of wound flux and two areas of background flux (normal skin perfusion) on opposite sides of the wound. Calculations comprised the fold change of Median Wound Flux/Median Background flux for each wound.

### 4.9. Histology and Pathological Analysis

Biopsy punches (8 mm diameter) were harvested on day 7 before reapplication of FSG and then on days 14, 21, 28, and 45 after all non-invasive measurements (digital images, LSI, TEWL, and hydration) were acquired. A strip biopsy spanning the entire wound bed from normal tissue through the middle of the wound to normal tissue was harvested at study completion on day 60. Biopsy samples were fixed in 10% neutral buffered formalin for at least 48 h and processed for paraffin embedding. Following processing, sections of tissue that were four micrometers thick were then cut and collected on a Superfrost^®^ Plus slide. Xylene was used to clear the slides, which were subsequently rehydrated using 100% ethanol, 95% ethanol, 70% ethanol, and DI water. For immunohistochemistry staining, the tissue sections were hydrated using Hank’s balanced salt solution (HBSS), blocked using 10% normal horse serum for 30 min, and incubated with the α-SMA antibody (1:100, AB7817, ABCAM, Cambridge, MA, USA) for 30 min at room temperature. Following incubation, the tissue was washed with HBSS, probed with a biotinylated secondary antibody, washed with HBSS, complexed with an avidin/HRP enzyme (Vectastain Elite ABC kit, Vector Laboratories, Burlingame, CA, USA), and detected with a DAB substrate kit (3,3′-diaminobenzidine, Vector Laboratories). Sections were then counterstained with Gill’s Hematoxylin, dehydrated, and mounted. For H&E’s, each section underwent standard staining. All slides were scanned via a Zeiss Axio Scan.Z1 (Zeiss, Oberkochen, Germany) at 20X and were exported as JPEGs. A board-certified veterinary pathologist blindly scored the slides based on the scoring system in Table 1.

### 4.10. Statistical Analysis

For contraction, re-epithelialization, and LSI, statistically significant differences were determined by two-way repeated measures, ANOVA with Tukey or Sidak’s post-hoc test, for multiple comparisons using GraphPad Prism 8.2.1 (GraphPad Software Inc, La Jolla, CA, USA). For TEWL and hydration, a 2 way ANOVA mixed effects model with Tukey post-hoc test was performed in GraphPad due to missing data points. All data are reported as the mean ± confidence interval (CI). Statistical significance was found when *p* < 0.05 and is indicated in the figures.

## 5. Conclusions

There is a growing need for cellular and tissue based products that can be used to treat severe burn wounds in austere conditions, particularly in situations where donor sites are scarce. This study demonstrates the superior wound healing properties of acellular FSG over its commercial comparator (FBD) in a preclinical porcine DPT burn wound model. Our findings support the use of FSG for achieving enhanced wound closure as evidenced by quicker integration and reepithelization without increased contraction. Though further evaluation is needed, this study indicates FSG’s potential as a clinically advantageous therapeutic for the temporary coverage of DPT burn wounds after NSD.

## Figures and Tables

**Table 1:** Pathology Scoring System.

Epithelial Status:	Foreign Material/Hemorrhage:
Score 0: None Score 1: Partial epidermis Score 2: 100% with hyperplasia Score 3: 100% Normal epidermis	Score 0: Absent Score 1: Present
Angiogenesis:	Hemorrhage Severity:
Score 0: None Score 1: ≤ 50 vessels Score 2: 51–100 vessels Score 3: 101–150 vessels Score 4: ≥ 151 vessels	Score 0: Absent Score 1: Mild Score 2: Moderate Score 3: Severe
Granulation Tissue Formation and Fibroplasia:	Neutrophils, Eosinophils, Lymphocytes, Macrophages:
Score 0: None Score 1: Minimal Score 2: Mild Score 3: Moderate Score 4: Marked Score 5: Severe	Score 0: None Score 1: Minimal # if inflammatory cells Score 2: Mild # of inflammatory cells Score 3: Moderate # of inflammatory cells Score 4: Marked # of inflammatory cells Score 5: Severe (high) # of inflammatory cells

## Data Availability

Due to the size of the raw files, datasets are available upon request.

## Supplementary Materials

Supplementary Materials can be found at https://www.mdpi.com/1422-0067/22/4/1590/s1.

## Abbreviations

α-SMA	Alpha smooth muscle actin
CBC	Complete blood count
CI	Confidence interval
CTP	Cellular and tissue-based product
DPT	Deep partial thickness
Dx	Normalized tattoo
FBD	Fetal bovine dermis
FDA	Food and Drug Administration
FSG	Fish skin graft
FT	Full thickness
H&E	Hematoxylin and eosin
IACUC	Institutional Animal Care and Use Committee
IM	Intramuscular
LSI	Laser speckle image
NSD	Non-surgical debridement
OWA	Open wound area
PFC	Prolonged field care
POSAS SQ	patient and observer scar assessment scale Subcutaneous
TBSA	Total body surface area
TEWL USAISR	Transepidermal water loss U.S. Army Institute of Surgical Research
