# Supplementary material for: Accelerated Wound Closure of Deep Partial Thickness Burns with Acellular Fish Skin Graft
Source: Int J Mol Sci. 2021 Feb 4;22(4):1590. doi: 10.3390/ijms22041590 (PMC7915828; doi:10.3390/ijms22041590)
Supplement: Supplementary file 1 [file ijms-22-01590-s001.pdf]

International Journal of
Molecular Sciences

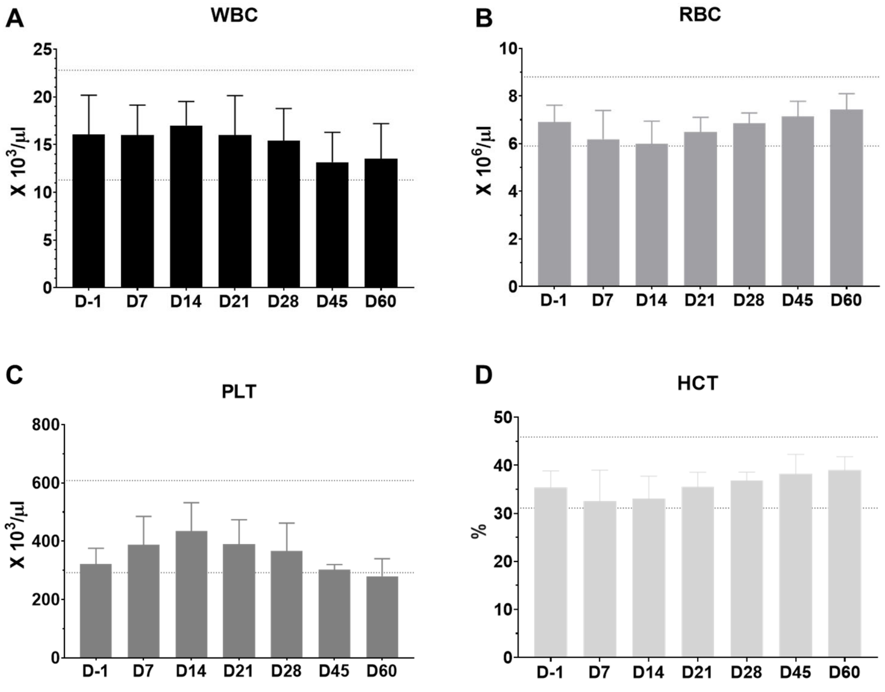

Supplemental Figure S1: Complete Blood Count Results**.** Average values for all 6 animals are shown throughout the experiment for A) white blood cells (WBC), B) red blood cells (RBC), C) platelets (PLT), and D) hematocrit (HCT). The mean with confidence intervals are illustrated here. The dotted lines represent the upper and lower normal values for porcine.

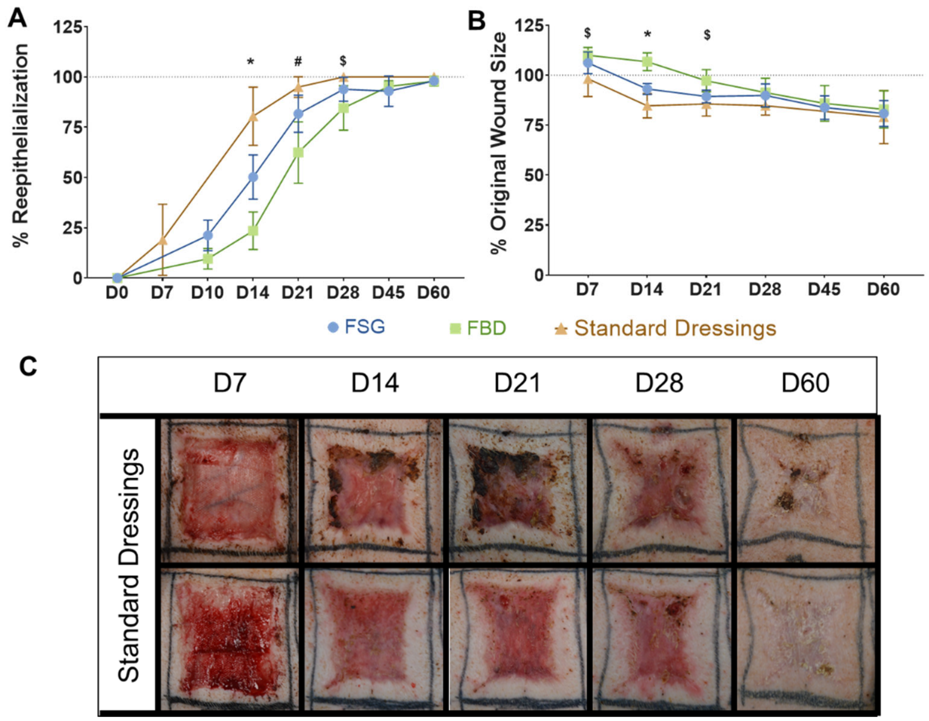

Supplemental Figure S2: Comparison to Historical Controls. A) Re-epithelialization was calculated by tracing the leading edge of the epidermis and comparing to total wound size (* = $p < 0.002$ comparing all groups; # = $p < 0.05$ CTPs vs. SD; $ = $p < 0.05$ for FBD vs. SD). Direct comparisons could not be made at Day 7, 10, or 45 because data collection did not coincide. Those time points were included on the graph above to illustrate the delay in the CTPs re-ep rate. B) Wound contraction was calculated by tracing the tattoos, comparing to the initial wound size, and normalizing to the growth of each animal (* = $p < 0.05$ comparing all groups; $ = $p < 0.05$ for FBD vs. SD).). Direct comparisons could not be made at Day 45 because data collection did not coincide. Significance for both A and B were determined by 2 way repeated measures ANOVA with Tukey post-hoc test. ($n = 12$ for FSG and FBD and $n = 5$ for SD). C) Representative digital images were captured throughout the study of wounds treated with standard dressings (antimicrobial Telfa) and were utilized to calculate the re-epithelialization and contraction rates. Biopsy punches are visible as small scabs in some wounds.

Epidermal Status

	FSG					FBD				
Path Score	D14	D21	D28	D45	D60	D14	D21	D28	D45	D60
0	7	4	1	1	0	10	6	3	0	0
1	4	4	0	2	1	1	4	1	1	0
2	1	4	10	8	8	1	2	8	9	10
3	0	0	1	1	3	0	0	0	1	2

Granulation Tissue/Fibroplasia

	FSG						FBD					
Path Score	D7	D14	D21	D28	D45	D60	D7	D14	D21	D28	D45	D60
0	0	0	0	0	0	0	2	0	0	0	0	0
1	0	0	0	0	0	0	6	1	0	0	0	0
2	3	2	0	0	4	0	4	5	1	0	5	1
3	7	3	2	5	6	3	0	6	4	5	3	3
4	2	7	5	7	2	8	0	0	5	6	3	8
5	0	0	5	0	0	1	0	0	2	1	1	0

Foreign Material

	FSG						FBD					
Path Score	D7	D14	D21	D28	D45	D60	D7	D14	D21	D28	D45	D60
0	3	7	12	12	12	12	4	2	7	10	12	12
1	9	5	0	0	0	0	8	10	5	2	0	0

Angiogenesis

	FSG						FBD					
Path Score	D7	D14	D21	D28	D45	D60	D7	D14	D21	D28	D45	D60
0	2	0	3	7	9	9	4	1	2	6	9	11
1	2	4	5	4	3	3	8	8	7	3	3	1
2	7	6	3	0	0	0	0	2	2	3	0	0
3	1	2	1	1	0	0	0	1	1	0	0	0
4	0	0	0	0	0	0	0	0	0	0	0	0

Hemorrhage Severity

	FSG						FBD					
Path Score	D7	D14	D21	D28	D45	D60	D7	D14	D21	D28	D45	D60
0	1	4	3	5	8	7	7	7	6	6	6	5
1	8	6	7	7	4	5	5	4	6	6	6	7
2	2	1	2	0	0	0	0	1	0	0	0	0
3	1	1	0	0	0	0	0	0	0	0	0	0

Neutrophils

	FSG						FBD					
Path Score	D7	D14	D21	D28	D45	D60	D7	D14	D21	D28	D45	D60
0	0	2	5	10	10	7	2	1	4	4	9	10
1	1	1	2	1	2	2	7	2	3	4	1	0
2	7	9	5	1	0	1	1	6	5	3	2	1
3	0	0	0	0	0	2	1	1	0	1	0	1
4	3	0	0	0	0	0	1	2	0	0	0	0
5	1	0	0	0	0	0	0	0	0	0	0	0

Eosinophils

	FSG						FBD					
Path Score	D7	D14	D21	D28	D45	D60	D7	D14	D21	D28	D45	D60
0	0	2	5	10	11	10	2	2	4	4	9	10
1	3	6	7	2	1	2	5	6	4	4	2	0
2	4	4	0	0	0	0	5	3	4	4	1	2
3	5	0	0	0	0	0	0	1	0	0	0	0
4	0	0	0	0	0	0	0	0	0	0	0	0
5	0	0	0	0	0	0	0	0	0	0	0	0

Lymphocytes

	FSG						FBD					
Path Score	D7	D14	D21	D28	D45	D60	D7	D14	D21	D28	D45	D60
0	0	1	2	8	9	5	1	0	1	1	4	1
1	0	2	4	2	3	2	5	2	2	1	2	2
2	9	8	6	2	0	3	6	8	3	5	4	5
3	3	1	0	0	0	0	0	2	5	4	2	3
4	0	0	0	0	0	2	0	0	1	0	0	0
5	0	0	0	0	0	0	0	0	0	1	0	1

Macrophages

	FSG						FBD					
Path Score	D7	D14	D21	D28	D45	D60	D7	D14	D21	D28	D45	D60
0	0	2	6	10	11	7	2	1	3	3	4	4
1	11	10	6	2	1	3	9	6	4	4	5	5
2	1	0	0	0	0	2	1	5	5	5	2	2
3	0	0	0	0	0	0	0	0	0	0	1	1
4	0	0	0	0	0	0	0	0	0	0	0	0
5	0	0	0	0	0	0	0	0	0	0	0	0

Supplemental Table S1. Pathology Scoring Results.
